# In Ethiopia’s Kutaber district, does community-based health insurance protect households from catastrophic health-care costs? A community- based comparative cross-sectional study

**DOI:** 10.1371/journal.pone.0281476

**Published:** 2023-02-15

**Authors:** Ayesheshim Muluneh Kassa

**Affiliations:** Department of Nursing, Dessie Health Science College, Dessie, Ethiopia; University of Bologna, ITALY

## Abstract

**Objective:**

Every health system needs to take action to shield households from the expense of medical costs. The Ethiopian government implemented community-based health insurance (CBHI) to protect households from catastrophic health care expenditure (CHE) and enhance the utilization of health care services. The impact of CBHI on CHE with total household expenditure and non-food expenditure measures hadn’t been studied, so the study aimed to evaluate the impact of CBHI on CHE among households in Kutaber district, Ethiopia.

**Methods:**

A total of 472 households (225 insured and 247 uninsured) were selected by multistage sampling techniques. Households total out-of-pocket (OOP) health payments ≥10% threshold of total household expenditure or ≥40% threshold of household non-food expenditure categorized as CHE. The co-variants for participation in the CBHI scheme were estimated by using a probit regression model. A propensity score matching analysis was used to determine the impact of CBHI on CHE. A Chi-square (χ2) test was computed to compare CHE between insured and uninsured households.

**Results:**

The magnitude of CHE was 39.1% with total household expenditure and 1.8% with non-food expenditure measures among insured households. Insured households were 46.3% protected from CHE when compared to uninsured households with total household expenditure measures and 24.2% to 25% with non-food expenditure measures.

**Conclusion:**

The magnitude of CHE was lower among CBHI-enrolled households. CBHI is an effective means of financial protection benefits for households as a share of total household expenditure and non-food expenditure measures. Therefore, increasing the upper limits of benefit packages, minimizing exclusions, and CBHI scale-up to uninsured households is essential.

## Introduction

Globally, about 44 million households face catastrophic health expenditures. From these, 25 million households were pushed into poverty because of direct health care payments and over 90% of healthcare financial difficulties and their consequences have been occurring in Sub-Saharan African countries where resources are limited [1]. The sub-Saharan region has a high burden of disease and low government expenditure on health. Thus, implementation of community-based health insurance (CBHI) has emerged as a possible health financing mechanism in reducing out-of-pocket costs, particularly in areas where many people engage in informal work and rural residences in these countries [2]. Health insurance remains an imperative policy strategy for improving health outcomes at a decisive time when many countries are pursuing the third Sustainable Development Goal (SDG) of safeguarding healthy lives and promoting well-being for all ages by the year 2030. It is one of the strategies geared towards achieving universal health coverage in lower and middle-income countries (LMICS) [1]. In the absence of any form of health insurance, out-of-pocket payments for health care lead to decreased use of health services and lead to catastrophic health expenditures [3]. Rural households the utmost developing countries are excluded from the formal insurance system. In many LMIC and middle-income countries (MICs), direct out-of-pocket payments dominate healthcare financing. Such direct payments are inequitable and inefficient in financing healthcare services [4].

Catastrophic healthcare expenditure (CHE) refers to any expenditure for medical spending of a household that exceeds a certain level of capacity that can pose a threat to a household’s financial ability to maintain its subsistence needs. Total health expenditure of 10% or more from total household expenditure is often considered an indication of catastrophic health expenditure. World Health Organization (WHO) showed that health spending be viewed as catastrophic whenever it is greater than or equal to 40% of a household’s non-food income, i.e., income available after basic needs have been met [5]. But countries may demand to use a different cut-off point in setting their state health policies. The threshold at which health payments become catastrophic has ranged from 10% to 40% of either total household expenditure, non-food expenditure, or expenditure net of basic food needs.

The effect of out-of-pocket expenses for health care goes beyond catastrophic spending alone. Many people may decide not to use services simply because they cannot afford the direct costs [6, 7]. Poor households are likely to sink even further into poverty because of the adverse effects of illness on their earnings and general welfare [8–11]. People who cannot afford health care services engage in alternative coping strategies such as borrowing money, mortgaging or selling assets; selling livestock, and withdrawing their children from school [7, 12]. A study conducted in 2017 by the Ethiopian Development Research Institute showed poor households has had a serious financial shock in the past 15 months because of the illness of a household member. Among non-CBHI households, reporting loss of consumption or assets due to an illness shock was 78.5% in South Ethiopia, Oromia 69.5% and Amhara 63.6% [13]. Due to financial constraints for health, the Ethiopian Federal Ministry of Health began health care financing reform to improve and diversify resource mobilization for health and secure financial protection for its citizens by implementing health insurance schemes [14].

Ethiopia is under-financed when compared to the Sub-Saharan African region. Donors (49.9%)and out-of-pocket expenditure (34%) are the main sources of health care financing and the government only covers 15.6%. The share of the health budget from the total allocated budget is 11.1% [15, 16].

The Ethiopian CBHI scheme is categorized as a government-run program with community participation in scheme design, management, and supervision. The main source of funding for the scheme are mainly the premium contributions of memberships (which range from 350–1700 ETB annually based on family size and taxation rank) and about 25% of the total premium subsidy from the central government. While district and regional governments cover the costs of providing a fee waiver for the poorest population groups (about 10% of the total population), CBHI payments are not intended to be a savings plan; if households do not use medical services, they will not receive a return or interest on their premium; rather, the premium is an annual payment made to cover future medical costs; membership in the CBHI scheme is voluntary and based on rural communities and the informal sectors at the household level. The CBHI scheme benefit packages include both outpatient and inpatient health-care services in public facilities, but it covers neither treatment outside the country nor medical treatment with largely cosmetic value. CBHI members are expected to first visit a health center, which can offer referral letters to higher-level care at district or regional hospitals as needed [3, 17].

CBHI brings down health care costs and better access to modern health services among insured households. However, there is moral hazard behavior among insured households–over-utilization of medical services. In Ethiopia there are no strong moral hazard prevention mechanisms (co-payment, co-insurance, deductibles, etc.),but the government used strong referral linkage as a protective mechanism [17]. There are some studies on the impact of the CBHI scheme on out-of-pocket payments of households in Ethiopia. But, evidence on the impact of CBHI on catastrophic health expenditure in Ethiopia by using the propensity score matching method at 10% threshold total household expenditure and 40% threshold non-food expenditure has not been done as far as my knowledge is concerned. The aim of this study is to assess the magnitude of catastrophic healthcare expenditure among insured and uninsured households and to evaluate the impact of CBHI on catastrophic healthcare expenditure. Therefore, this study will provide additional knowledge for policy makers and planners. It will be used as a baseline for further research for academicians and researchers. The Ethiopian health insurance agency will also use it as an evaluation of the CBHI scheme.

## Materials and methods

### Study settings

The study was conducted in Kutaber district, South Wollo Zone, Amhara National Regional State, Ethiopia. As reported by the District Health Office (Annual Report of Kutaber District Health Office, 2020, unpublished), Kutaber has 23 kebeles (22 rural, 1 urban), 5 public health centers, 23 health posts, and 3 drug vendors. The total population was 109,746, of whom 56,366 were males and 25,522 households, of which 13,980 were enrolled in the CBHI scheme. CBHI member households who had all lived in the rural area of the selected “kebeles” (the lowest administrative unit in Ethiopia with a population of around 5000) for more than 1 year and willingly gave consent to being included in the study. There were 48 health extension workers who worked as front-line health service providers.

### Study design and period

A community-based comparative cross-sectional study was conducted to evaluate the impact of community-based health insurance on catastrophic health care expenditure in Ethiopia from June 01 to 27, 2020.

### Participants

The source of the population were households in the rural community of Kutaber district, Amhara Regional State, Ethiopia. The study population were households with CBHI and non-CBHI members found in the selected kebeles of the Kutaber district.

### Inclusion and exclusion criteria

Permanent residents of the community with the household head/spouse/available during the study period but for CBHI members one year and above after enrolling were included in the study.

Households with a critically ill head/spouse during the study period and new CBHI members (less than a year since enrollment) were excluded from the study.

### Sampling procedures

A two-stage sampling method was used to select study participants. I included a total of 494 households in the study; I used a simple random sampling technique at each stage to eliminate selection bias. In the first stage, five kebeles were selected (Alasha, Doshegne, Beshelo, Haruye and Kundie) out of a total of 23 (22 rural and 1 urban) kebeles using a lottery method. In the second stage, using data obtained from Kutaber district CBHI coordinating office, households insured and uninsured in the selected kebeles were identified using their family folder number and individual registration identification numbers from the registration book through the support of health extension workers. Then, the sample size was proportionally allocated based on insurance status (insured and uninsured) to each sampled kebele.

### Sample size determination

The sample size for this study was calculated using the double population proportion formula.The estimation of the sample size was done by assuming a study conducted in Tehulderie district, p1 = 15.64% of uninsured households and p2 = 4.41% of insured households were affected by catastrophic healthcare expenditure, 95% confidence level and 5% margin of error [2]. The calculated sample size for this study was checked by Epi-Info version 7; the sample size was 224 from insured households and 224 from uninsured households. Tolerable non-response rate was 10%; hence, the sampling procedure was multistage sampling: the first stage was kebeles, and then the second stage was households within the kebeles; and a design effect of two was used. The total sample size was 494 households (247 insured and 247 uninsured households). Then, the sample size was proportionally allocated based on insurance status (insured and uninsured) to each sampled kebele. The insured to uninsured ratio was 1:1.

### Data collection

The questionnaire for this study was adapted from the Ethiopian health insurance agency household survey questionnaire [17] and from literatures developed for similar purposes by different authors and reviewed to suit the local situation [9, 18, 19]. Face-to-face interviews with household heads were conducted using a pretested, structured questionnaire that was prepared in English and then translated into Amharic by a language expert. Then, it was re-translated back to English to check for conceptual equivalence. The questionnaire covered socio-demographic variables (age, sex, marital status, religion of household head/spouse and family size), socio-economic variables (wealth index, education, CBHI status and occupation of household head) and health and health- related variables (chronic illness, acute illness, health status of household, under 5 children, pregnant in the last year, travel time, type of health facility used, accidental injuries and over 50 year old household members). The validity of the tool was checked by a group of experts (health economists, behavioral scientists, and epidemiologists). The data was collected by ten health extension workers, and the data collection process was supervised by two bachelor-level nurses. A day of training had been given to the data collectors and the supervisors prior to the data collection process.

### Operational definitions

Out-of-pocket (OOP) are payments paid by households to the point they receive health services (consultation fees, drugs, hospital bills, traditional medicine, loading/board/food/shelter/and transportation at the household level for the last 12 months for health were included, both inpatient and outpatient). Total household expenditure (THEH) food expenditure was estimated at the household level from purchased foodstuffs and food from the harvest or stock spent and loaned for the last 7 days. Nonfood expenditure (NFEH) was estimated at the household level for the last 12 months from clothes and related expenditure; housing and related expenditure; social obligation; health expenditure; education expenditure; agricultural inputs; and livestock and death-related expenditure.

**Catastrophic healthcare expenditure (CHE): This** occurs when a household’s total OOP health payment ≥ 10% of the households’ total household expenditure[20] or ≥40% of the households’ non-food expenditure[21].

Households with CHE_H_

CHE_H_ = 1 if OOP_H_ ≥10% Total households’ expenditure

THE_H_

Households with CHE_H_

CHE_H_ = 2 if OOP_H_ ≥40% Non-food expenditure

NFE_H_

Household head: any person in the household who is recognized as the household head by the other members of the household. She/he is the person responsible for the maintenance and care of the household. Health status of the household: household head/spouses’ self-reported health condition of the household. Insured households those who are members of CBHI for more than or equal to one year after enrolling at the household level. Uninsured households: those who weren’t members of CBHI before and new CBHI members (less than a year since enrollment) at the household level. Chronic illness: household members who have diabetes, asthma, cardiac disorders, arthritis, HIV/AIDS, hypertension, ulcers, gout, cancer, and sinusitis. Acute illness: household members who have experienced any illness during the last three months. Accidental injuries: household members who have sustained bodily injuries like sudden falls, cuts, burns, road accidents, bites, stings, and drowning in the last year.

### Data quality assurance

Pre-tests were conducted on 5% of the total sample size in the district prior to the main study to identify potential problems in data collection tools and amendments to the questionnaire. The questionnaire was pre-tested on local people living outside the sampled kebeles. The questionnaires used in the pre-test would not be included in the analysis as part of the main study. Training on the subject matter of the study had been given to data collectors and supervisors before the day of data collection. The collected data was properly handled, reviewed and checked for completeness and consistency by the supervisor and principal investigator before commencing analysis each day.

### Data processing and analysis

EPI data version 4.6 was used to code and enter the data, and STATA version 14.2 was used to analyze it. The data was summarized using descriptive statistics. A probit regression model was used to identify co-variants that affected CBHI scheme participation. A propensity score matching analysis was used to assess the impact of community-based health insurance on catastrophic healthcare expenditure.

Total household expenditure was calculated as food expenditure, which was estimated at the household level from purchased food staff and food from harvest or stock spent and loaned for the last 7 days. Then all these expenditure-related variables were converted to monthly figures plus non-food expenditure. Nonfood expenditure was estimated at the household level for the last 12 months from clothes and related expenditure, housing and related expenditure, social obligations, health expenditure, education expenditure, agricultural inputs and livestock, and death-related expenditure. Then all these expenditure-related variables were converted to monthly figures. CHE is calculated as the burden of OOP health expenditure as a nominator, from total household expenditure as a denominator at a 10% threshold, or OOP from non-food expenditure as a denominator at a 40% threshold categorized as catastrophic health expenditure. OOP payments paid by households at the point they received health services, consultation fees, drugs, hospital bills, traditional medicine, loading, board, food, shelter, and transportation for health, both inpatient and outpatient, for the last 12 months, and all these expenditures converted to monthly figures were included in the data analysis.

The comparison of households’ characteristics among insured and uninsured households, t-value with 95% CI, was computed using two-sample (independent sample) t-tests.

A chi-square (χ2) test was used to compare catastrophic health expenditure between insured and uninsured households, and cronbach’s alpha was used to assess tool reliability. The probit regression model is a method of fitting to compare the relationship of enrolling in the CBHI scheme to the co-variants. Those variables that affect participation in the scheme are selected and generated for PSM analysis. The propensity matching method constructs a statistical comparison group that is based on a model of the probability of participating in the treatment T conditional on observed characteristics X, or the propensity score: (T = 1|X) P (X) = PR (T = 1 |X). P (X) = PR (T = 1 |X) This shows that, under certain assumptions, matching on P (X) is as good as matching on X. Enrolled participants are then matched on the basis of this probability, or propensity score, to non-enrolled. The average treatment effect of the CBHI is then calculated as the mean difference in CHEs across these two groups. The necessary assumptions for the identification of the CBHI effect are conditional independence and the presence of common support. Conditional independence states, which give a set of observable covariates X that are not affected by treatment, and potential outcomes Y are independent of treatment assignment T. This assumption is also called un-confoundedness, and it implies that uptake of the CBHI is based entirely on observed characteristics. If unobserved characteristics determine CBHI participation, conditional independence will be violated, and propensity matching is not an appropriate method. Another assumption is the common support or overlap condition: 0 < P (Ti = 1|Xi) < 1. This condition reveals that treatment of the treated has a comparison to the untreated “close” in the propensity score distribution. Specifically, the effectiveness of propensity score matching also depends on having a large and roughly equal number of insured and uninsured observations so that a substantial region is common. The first matching technique is the nearest-neighbor, in which each treated unit is matched to the unit in the comparison group that presents the closest estimated propensity score. After matching, the result of the treated units is compared with the result of matched control units. The second matching method is caliper matching, which holds matching with replacement only among propensity scores within a certain range. The third matching technique is kernel matching, which assigns higher weight to observations close in terms of propensity score to a treated individual and lower weight to more distant observations.

### Ethical approval

The study protocol was obtained from Wollo University, Institutional Review Board (Ref. No. 320/2020).Verbal informed consent was obtained after the orientation of the subjects concerning the objectives of the study, the data confidentiality and the importance of the study. The data collectors were reading informed consent to study participants who were not able to read and write. Study participants who could be able to read were offered to read the informed consent sheet. Participants who were unwilling to participate in the study and those who wished to quit from the study at any point in time were informed to do so without any restriction. This study conducted in accordance with the Declaration of Helsinki.

## Results

### Demographic and socio-economic characteristics among insured and uninsured households

The majority of the respondents were male, both among the insured (91.11%) and uninsured (84.21%). Age, sex, education, religion, family size, and wealth index showed significant differences between the insured and uninsured households, but the variables’ marital status and occupation did not show disparity between the insured and uninsured households **(Table 1).**

**Table 1:** Demographic and socio-economic characteristics of insured and uninsured households in Kutaber district, Ethiopia, 2020 (n = 472).

Variable	Community based health insurance			
	Insured n (%)	Uninsured n (%)	Different	P- value
**Age of household head/Spouse /**				
25–35	60(26.67)	87(35.22)	-8.55	0.027*
36–45	87(38.67)	72(29.15)	9.52	0.015*
46–55	33(14.67)	36(14.57)	0.1	0.333
56–65	21(9.33)	27(10.84)	-1.51	0.720
>65	24(10.67)	25(10.04)	0.63	0.318
**Sex of household head/Spouse**				
Male	205(91.11)	208(84.21)	6.9	0.025*
Female	20(8.89)	39(15.79)	-6.9	0.015*
**Marital status of household head/Spouse**				
Single	14(6.22)	11(4.45)	1.77	0.549
Married	190(84.44)	205(82.99)	1.45	0.445
Others¥	21(9.33)	31(12.55)	-3.22	0.200
**Religion of household head /Spouse**				
Orthodox	46(20.44)	76(30.77)	-10.33	0.007*
Muslim	179(79.56)	171(69.23)	10.33	0.011*
**Wealth index household**				
Poor	117(52.00)	205(82.73)	-30.73	<0.0001*
Medium	30(13.33)	21(8.43)	4.9	0.001*
Rich	70(34.67)	21(8.84)	25.83	<0.0001*
**Educational status of household head/Spouse**				
No formal	129(57.33)	180(73.09)	-15.76	<0.0001*
Formal	96(42.67)	67(26.91)	15.76	0.004*
**Family size of household**				
**<5**	76(33.78)	128(51.81)	-18.03	<0.0001*
**> = 5**	149(66.22)	119(48.19)	18.03	<0.0001*
**Occupation of household head/Spouse**				
Farming	194(86.22)	203(81.53)	4.69	0.156
Non farming	31(13.78)	46(18.47)	-4.69	0.089

Others ¥: divorce, widow *Statistically significant at p<0.05

Forty-five percent of the uninsured and 35.11% of the insured respondents were identified as having poor health status. The study variables, family member over the age of 50, type of health facility, time traveled in hours, and accidental injuries in the previous year differed significantly between insured and uninsured households. However, children less than five years old in the household, a history of illness in the past 3 months, and chronic illness in the family were not significantly different from enrollment in community-based health insurance by households **(Table 2).**

**Table 2:** Health and health- related factors among insured and uninsured households in Kutaber district, Ethiopia, 2020 (n = 472).

Variables	Community based health insurance				
	Insured n (%)	Uninsured n (%)		Difference	P-value
**Perceived health status of household**					
Poor	79(35.11)		111(44.94)	-9.83	0.006*
Medium	53(23.56)		64(25.91)	-2.35	0.068
Good	93(41.33)		72(29.15)	12.18	0.103
**Chronic illness in the family**					
Yes	32(14.22)		30(12.15)	2.07	0.505
No	193(85.78)		217(87.85)	-2.07	0.236
**Children less than five years in the household**					
Yes	99(44.00)		104(42.11)	1.89	0.678
No	126(56.00)		143(57.89)	-1.89	0.300
**Family member above 50 years**					
Yes	71(31.56)		118(47.78)	-16.22	<0.0001*
No	154(68.44)		129(52.22)	16.22	0.138
**History of illness in the past 3months**					
Yes	153(68.00)		168(68.02)	0.99	0.997
No	72(32.00)		79(31.98)	0.02	0.569
**Type of health facility**					
Public	184(81.78)		141(57.09)	24.69	0.017*
Others††	41(18.22)		106(42.91)	-24.69	<0.0001*
**Time travel in hours**					
<1	77(34.22)		47(19.03)	15.19	0.008*
> = 1	148(65.78)		200(80.97)	-15.19	<0.0001*
**Pregnancy in the last year**					
Yes	35(15.56)		55(22.27)	-6.71	0.065
No	190(84.44)		192(77.73)	6.71	0.918
**Accidental injuries in last year**					
Yes	32(14.22		53(21.46)	-7.24	0.042*
No	193(85.78(		194(78.54)	7.24	0.959

Others

†† private, traditional healers

*Statistically significant at p<0.05.

Among households that faced catastrophic healthcare expenditure, (89.5%) were uninsured and (39.1%) were insured households by total household expenditure measured at the 10% threshold level. Of the studied households, 1.8% insured and 26.7% uninsured incurred catastrophic health expenditure by non-food expenditure measured at the 40% threshold **(Table 3).**

**Table 3:** Catastrophic health care expenditure measured as a percentage of total household expenditure and non-food expenditure at 10% and 40% thresholds among insured and uninsured households in Kutaber district, Ethiopia, 2020(n = 472).

Level of health Insurance status of households (n)	Type of catastrophic health care expenditure							
	10% of total household Expenditure				40% of Non-Food Expenditure			
	Yes (n, %)	No (n, %)	X^2^	P- value	Yes (n, %)	No (n, %)	X^2^	P- value
Insured(n = 225)	88(39.1)	137(60.9)	108.78	<0.0001*	4(1.8)	221(98.2)	58.61	<0.0001*
Uninsured(n = 247)	211(89.5)	36(14.5)	Ref	Ref	66(26.7)	181(73.3)	Ref	Ref

*p value<0.0001

### Propensity scores matching analysis

The results showed that enrolling in the CBHI scheme had a 44-percent lower chance of incurring catastrophic health care expenditure than not enrolling in the CBHI scheme at the 10% threshold with the total household expenditure measures. In addition, the average treatment on the treated (ATT) catastrophic health care expenditure after matching revealed that joining in the CBHI scheme was 21 percent, reduce the chance of suffering catastrophic healthcare expenditure compared to those of not joining in the scheme at the 40% threshold by non-food expenditure measure **(Table 4).**

**Table 4:** ATT on insured and uninsured households by total household expenditure and non-food measures in Kutaber district, Ethiopia, 2020 (n = 472).

Variables	Sample	Treated	Controls	Difference	SE	T-stat
**CHCE1**	Unmatched	0.39	0.85	-0.46	0.04	-11.86
	ATT	0.39	0.83	-0.44	0.07	-6.12
**CHCE2**	Unmatched	0.02	0.27	-0.25	0.03	-8.11
	ATT	0.02	0.23	-0.21	0.07	-3.16

CHCE1 = Catastrophic health care expenditure by total household expenditure measures CHCE2 = Catastrophic health care expenditure by non-food expenditure measures.

Participation in a community-based health insurance scheme in Kutaber District was influenced by the wealth index, education, family size, type of health facility, old age, and travel time in hours. A one-member increase in family size increases the probability of CBHI enrollment status by 0.32. Similarly, participants from rich households increased the probability of CBHI enrollment status by 0.53. However, a one-year increase in age decreases the probability of CBHI enrollment status by -0.52. Likewise, participants who utilized private health facilities decreased the probability of CBHI engagement by -0.78 (**Table 5).**

**Table 5:** Probit regression of participation in a community- based health insurance scheme in the Kutaber district of Ethiopia, 2020.

Covariants	Coef.	Std.Err	Z
Sex	0.401827	0.2502343	1.61
Age	0.0424188	0.0659067	0.64
marital1	-0.0028953	0.1935424	-0.01
Education	0.3408488	0.157075	2.17*
Occupation	-0.0550452	0.2281219	-0.24
Type of health facility	-0.7840576	0.1406233	-5.58***
Perceived health status	0.1664489	0.0964319	1.73
Chronic illness	0.0256294	0.2069526	0.12
Old age	-0.5236746	0.1635223	-3.20***
Time travel	-0.3785855	0.1545693	-2.45*
Wealth index	0.5272149	0.0836327	6.30***
Family size	0.3227783	0.1374854	2.35*
Cons	1.457006	0.4422254	3.29***
LR chi2(7)	140.06		
Prob > chi2	0.0000		

*Statistically significant *P< 0.05, ***P <0.001 Source: Own survey result, 2020

**Fig 1:**
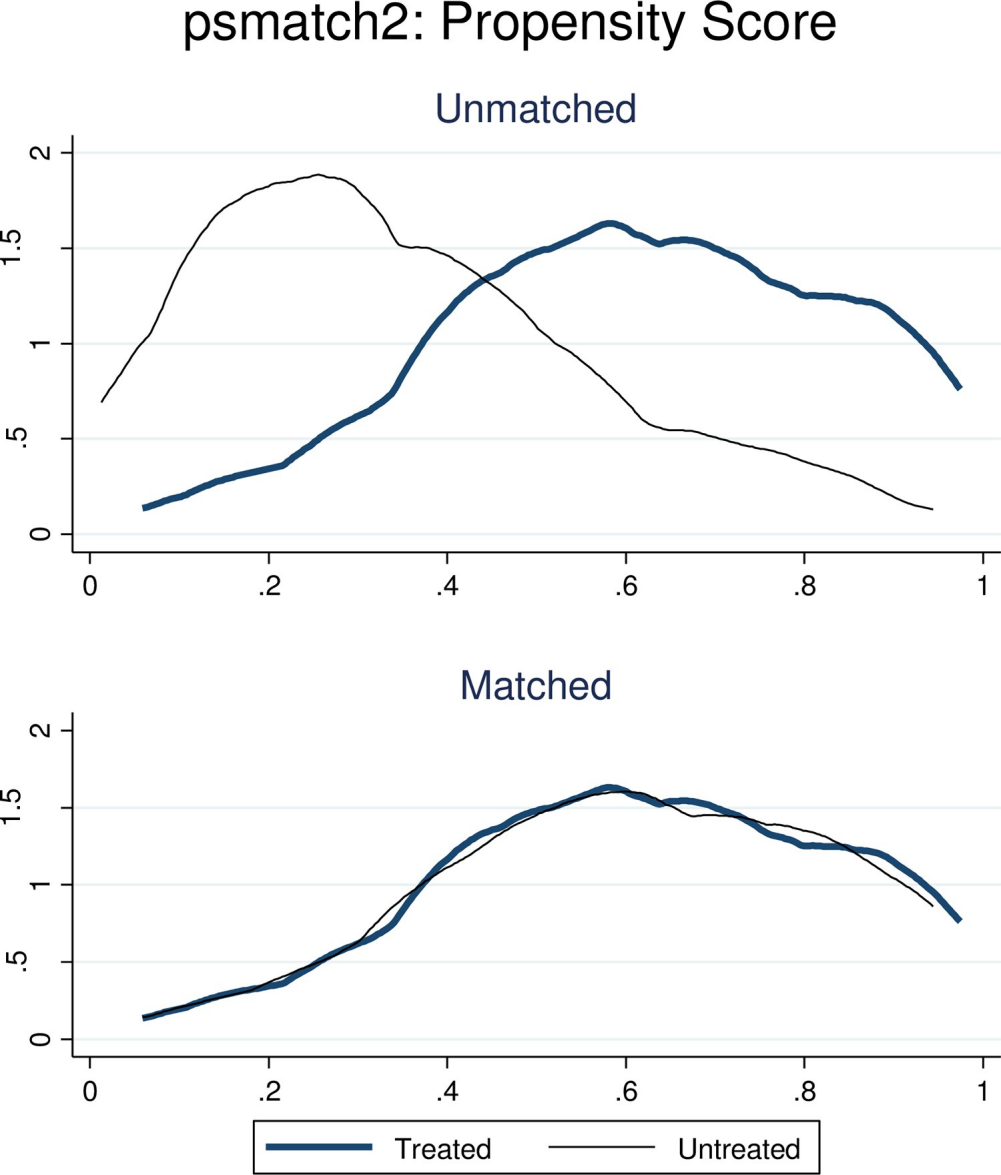
The comparison of the density estimation of both insured and uninsured groups before and after matching in Kutaber district, Ethiopia, 2020. The common support area is those propensity scores within the range of the lowest and highest estimated values for households in the treatment groups. Before the propensity score matching, the insured and uninsured groups were dissimilar with regard to the characteristics measured by the propensity score, and after matching, they were similar.

The estimated propensity score before matching of the treated households varied between 0.02 and 1.0, in which the mean propensity score was 0.505, and the estimated propensity score of control households ranged from 0.08 to 0.86, with the mean being 0.47. The total propensity score distribution of households after matching would lie between 0.02 and 1.0, with a mean of 0.505 **(Fig 1).**

In the balancing property all untreated and treated groups balanced by psmatch2, pstest, both graph, psgraph and pstest _pscore, density both. To check t-test pstest was done and the mean bias was less than 5%.

An estimation of the treatment effect of community-based health insurance using different matching techniques was done. It indicates a community-based health insurance scheme has a significant impact on households’ catastrophic health care expenditure, both in total household expenditure and non-food expenditure measures. The impact of CBHI on CHE was 46.3% lower among insured households than uninsured households. (t = -13.10), (95% CI: -0.53, -0.39) by kernel matching at 10% of the threshold level with total household expenditure measures. In the meantime, the average CBHI impact on catastrophic health care expenditure among insured households is 25% lower than uninsured households at 40% of the threshold level with non-food expenditure measured by nearest neighbor and kernel matching methods (t = -6.13.4), (95% CI: -0.33, -0.17). Therefore, estimations using the three methods below indicate that enrolling in the CBHI scheme significantly reduces catastrophic healthcare expenditure **(Table 6).**

**Table 6:** The results of the average treatment effects of community-based health insurance on outcome variables were used by using different propensity score matching algorithms in Kutaber district, Ethiopia (n = 472).

Outcome variables	Treated variable	Matching Methods	ATT	S.E.	T-stat	95%CI
CHCE1 at 10%	Treated 225	Nearest neighbor Matching	-0.463	0.040	-11.61	-0.542, -0.383
	Controls 247	Radius matching	-0.463	0.038	-12.30	-0.538, -0.388
		Kernel Matching	-0.463	0.035	-13.10	-0.533, -0.393
CHCE2 at 40%	Treated 225	Nearest neighbor Matching	-0.250	0.043	-5.87	-0.334, -0.166
	Controls 247	Radius matching	-0.242	0.029	-8.38	-0.300, -0.185
		Kernel Matching	-0.250	0.041	-6.13	-0.331, -0.169

***Notes*:** *number of replications = 100*. *ATT–average treatment of treated; SE-standard error; CI-confidence interval*

## Discussion

The aim of this study was to assess the magnitude of CHE among insured and uninsured households and to evaluate the impact of CBHI on CHE in Kutaber district, Ethiopia. The magnitude of catastrophic health care spending was lower among insured households compared to uninsured ones. Even though propensity score matching has a weak causal-effect relationship, the analysis showed that insured households were protected from catastrophic health expenditure both by total household expenditure and non-food expenditure measures.

In this study, the magnitude of catastrophic healthcare expenditure at the 10% threshold of total household expenditure was 39.1% among insured households when compared to uninsured households (89.5%). This result is higher in magnitude than similar studies conducted in Kenya (10.2%) [22], Ghana (18%) [5], Cote d’Ivoire (3.9) [23], India (23%) [24], China (14.4%) [25]. South Africa (25%) [4, 5], Nigeria (16.4%) [8], UK (0.04%), France (0.01%) [9, 11], Kosovo (15%) [6], South Korea (4.1%) [9], Iran (22.2%) [10], Myanmar (21%) [13], Tehran 29.9% [14], and LMICS (29%) [15].

A number of reasons have been cited to explain these inconsistent findings. For example, the health benefit package of national hospital insurance in Kenya covers many health packages (example, renal dialysis, kidney transplant; radiology package (MRI and CT scan); oncology package (cancer treatment, chemotherapy, radiotherapy, rehabilitation, drug and substance abuse package; foreign treatment packages etc.) However, such health benefit packages to cover household health care costs are not covered by Ethiopia’s community-based health insurance scheme. Moreover, there is a high burden of communicable disease in the study area, which makes the households forego frequent visits to health facilities, leading to worsening indirect costs (loading, board, food, shelter, and transportation) and socio-economic differences among countries.

Furthermore, unaffordable health care cost (access to private health facilities), scarce resources among Ethiopian households, a significant large proportion of the Kutaber population living just below the poverty line for them, an even lower amount of OOP health expenditure for services not covered by the insurance scheme result in catastrophic health care costs and impoverishment [26]. Dropping such disparities is one of the important objectives for achieving universal health coverage.

But,this finding was lower than a study done in Ghana(56%-87%) [27]. This might be due to the health care services that are not covered under the national health insurance scheme packages in Ghana (for example, appliances and prostheses including optical aids, hearing aids, orthopaedic aids and dentures, reconstructive surgery, HIV antiretroviral medicines, echocardiography, orthoptics, heart and brain surgery, cancer treatment other than cervical and breast cancer, and mortuary services, etc.) requiring out-of-pocket payments, most of which are incorporated into Ethiopia’s CBHI scheme.

The impact of CBHI status (being insured households) using propensity score matching was protected from catastrophic health expenditure by 46.3% at the 10% threshold level in different matching algorithm methods. The protective effect of insurance against catastrophic health care expenditure is particularly strong among CBHI members compared to their comparison groups in all measures. This finding is supported by a study done in Tanzania which revealed that insured households have a 15.1% reduction in catastrophic health expenditure. In Rwanda it was 17% [18] and, in Ghana, 37% protected catastrophic payments using 10% threshold household expenditure among insured households [27]. Other research conducted in developing countries, such as Rwanda, Latin America, Ghana, and Bangladesh, indicates that CBHI has a protective effect against catastrophic healthcare expenditure [7, 18, 28–31]. Contrary to this finding, studies done in South Africa, China, Rwanda, and Sudan showed that CBHI had no effect on financial protection [32–35]. This might be due to differences in socio-economic status, methodological differences in the measurement of CHE, benefit packages, and the by-pass of government health facilities to high-quality health care centers.

Using the standard WHO approach, this finding showed that the magnitude of catastrophic healthcare expenditure at the 40% threshold of non-food expenditure measures was 1.8% among the insured compared to the uninsured (26.7%). This finding was lower in studies done in Togo (9.7%) [36], Kenya (6.6%), Zambia (11.2%), Malawi (0.7) [8], Ghana (2.43%) [37], Nigeria (13.7%) [8], Nepal (25%), Bangladesh (44%) [28, 38], Kosovo (25%), Serbia (37.9%) and Macedonia (23%) [6]. CBHI in Ethiopia is a comprehensive benefit package with a low level of OOP at a 40% threshold, no co-payment and co-insurance mechanisms. In addition, it might be due to socioeconomic and cultural differences, inaccessible health institutions, study design, and sample size. The propensity score matching results indicated that CBHI status (being insured households) decreases catastrophic health expenditure by 25% at a 40% threshold level using nearest neighbor and kernel matching algorithms. This finding was similar to a study done in Ethiopia in which insured households had a lower probability of incurring catastrophic health expenditure by 23.2% [17]. Other studies done in developing countries, such as Ghana and Saudi Arabia, showed comparable results, with CBHI contributing to reducing CHE among members joined in the scheme relative to those not joined in the scheme [22, 27, 39]. But contrary to this finding a study done in rural India found that CBHI has no effect on catastrophic health expenditure [12]. Many reasons have been cited to explain these paradoxical findings. First, it might be due to the low benefit of the CBHI package, which results in households spending out of pocket from their side. Second, due to the lack of efficient risk pooling mechanisms, they face higher poverty rates, and it might be due to the fact that being covered under a health insurance system didn’t guarantee protection from facing CHE. Third, due to the difference in sample size and socio-economic status of the study households.

A study done in Ghana protects 48% of the insured households from catastrophic healthcare expenditure by using 40% of subsistence expenditure [5]. Other study done in Nigeria was shown to reduce annual health expenditure by $3.80–138 on average. Health insurance coverage affects total OOP expenses by accessing health care services and potentially reducing financial risks [8]. The findings are consistent with other results in India and the literature, which show CBHI reduces the financial burden for households [40]. Generally, CBHI ensures financial protection for households to mitigate against catastrophic health care expenditure and poverty induced by out-of-pocket.

The policy implications of this study reveal that the government of Ethiopia needs to either scale up the current community-based health insurance to uninsured households or establish a new financial protection mechanism for the households by developing supportive strategies. Moreover, creating awareness and community mobilization on the importance of being a member of CBHI and renewing households’ membership regularly is essential. This study is important for policymakers to evaluate the CBHI program in Kutaber district. Being I have no baseline data and no cut-off point to enroll in the CBHI, I used propensity score matching, which ignores the effects of unobserved characteristics and weak cause-effect relationships. Besides, the sample size is small, which may have an effect on the results of the study.

### Conclusions

The magnitude of catastrophic health care spending was lower among insured households than among uninsured households. Community-based health insurance protects households from catastrophic health expenditure as a share of total household expenditure and non-food expenditure measures. Policymakers and planners should redouble their efforts to ensure that everybody is covered by a pre-payment system like the national health insurance scheme in Ethiopia.

I recommended it to other researchers to do the same with other methods like difference-in-difference, regression discontinuity design, instrumental variable, and endogenous switching regression.
